# Supplementary material for: Identification of risk factors and mosquito vectors associated with dengue virus infection in American Samoa, 2017
Source: PLOS Glob Public Health. 2023 Jul 7;3(7):e0001604. doi: 10.1371/journal.pgph.0001604 (PMC10328243; doi:10.1371/journal.pgph.0001604)
Supplement: Attachment — Submitted filename: Response to reviewers comments_12-13-2022.docx [file pgph.0001604.s003.docx]

**Response to Reviewers’ Comments:**

**“Identification of Risk Factors and Mosquito Vectors associated with Dengue Virus Infection through Household-based Cluster Investigations in American Samoa” (PGPH-D-22-01116)**

Reviewer #1: The study entitled, "Identification of Risk Factors and Mosquito Vectors associated with Dengue Virus Infection through Household-based Cluster Investigations in American Samoa" is a simple study, which is based on questionnaire, and RT-PCR and anti-DENV IgM ELISA techniques. Although the study is a basic study but is well designed and effectively executed. However, the introduction section is very short and poorly framed. The rationale behind the study and the key questions of the research are not very clear. I think the authors need to reframe the introduction part. Moreover, in the methodology part, the authors need to mention a brief protocol (or the citation) for the identification of the mosquito species. Rest of the manuscript is ok.

*We thank the reviewer for this feedback. We have added additional information to the Introduction to better contextualize this investigation. We have also added additional detail to the Methods to clarify the approach used for mosquito identification.*

Reviewer #2: Authors have described a household-based cluster investigation to identify population-specific risk factors associated with infection and performed entomologic surveillance to determine the relative abundance of Ae. aegypti and Ae. polynesiensis. The manuscript is well written and worth of publication at PLOS GPH, after minor revision.

It’s ok to use either mosquitos or mosquitoes, but please standardize throughout the manuscript. Additionally, some typing errors when writing Aedes species. Please review all manuscript carefully.

*We appreciate this point and have replaced “mosquitos” with “mosquitoes” throughout the manuscript.*

Author Summary.
Line 56 – Please add a space “some Aedes species…”

*Done.*

Line 62- Please correct. In the abstract and results section authors mentioned seven DENV-positive cases (ELISA = 7 and RT-PCR=2), but here says nine.

*Done.*

Materials and methods
Authors should add a space between number and the IU (celsius, min, etc.)

*Done.*

Results
I really recommend authors to provide a map showing the positive cases. Is there any proximity between locations? Any patterns?

*We appreciate this suggestion. However, GPS coordinates were not routinely available from all cases, which prohibited analysis of geospatial trends of cases.*

Page 13, Lines 231-2 – Authors stated, “The percentage of febrile illness attributable to DENV infection was 48.1%.”
What are others febrile illness reported in the Island? It will be important to discuss more this finding.

*While we agree with the reviewer’s comment, to our awareness studies have not been conducted to comprehensively enumerate the most common causes of acute febrile illness in American Samoa. Moreover, the relative proportions of each disease would not be expected to be temporally generalizable (e.g., 48% of fevers being attributable to DENV infection would be expected to be higher than normal due to the dengue outbreak occurring at the time of the investigation).*

Reviewer #3: In this manuscript the authors present the results of a household-based cluster investigation that was conducted during the 2017 outbreak of dengue fever in the American Samoa. The author’s findings are interesting in that they suggest that a significant number of containers of standing water were found that could be used for breeding, and indeed a considerable number were found to have pupae present in them. The linkage between the presence of a septic tank is also interesting, although as the author’s note, the sample size does limit the statistical power. Overall, the manuscript is well written, and the statistical analysis contained sufficient detail that I have only minor comments about the overall manuscript.

-- Minor Points --
While the authors provide some overview of the literature concerning the linkage between dengue and septic tanks in the discussion (starting at Line 271), it may be worth pointing to some of this literature in the introduction for those researchers that are still in the process of getting read-up on the dengue literature.

*We appreciate this suggestion and have added text to the Introduction accordingly.*

With regards to the above point, since the sample size is small enough, it might be worth noting the type of septic tank if that was recorded.

*Unfortunately, the specific type(s) of septic tanks was not collected during the household surveys.*

Minor typo in Table 2: lemon grass / limon grass

*Corrected.*

Reviewer #4: Congratulations, Although there were problems with the sample size, the paper is the result of a detailed analysis of the data and with potential for future projects.

Methods

Ethics statement: All research that involve human beings and collection of blood, tissues etc, needs an ethical approval. Do you have one?.

*As documented with the journal and summarized in the Methods, the protocol for this investigation was not subject to IRB review as it did not meet the definition of ‘research’.*

Household-based cluster investigations:
Please clarify if the patients reported with a suspected dengue (index cases), were tested again to dectect DENV, CHIKV and ZIKV.

*As stated in the Methods, all participants of household-based cluster investigations were tested for evidence of acute infection with DENV, CHIKV, and ZIKV by RT-PCR and for recent infection with DENV by anti-DENV IgM ELISA, regardless of if they had been previously identified as an acute dengue case.*

Entomologic surveys:
The pupae or larvae you found in water-holding containers were collected to be identified at the laboratory? Please clarify

*Immature mosquitoes were not collected as a part of this investigation. This detail has been specified in the Methods.*

Results

You worked with 21 index cases? And then removed 2? Please clarify this.

*Correct. As stated in the Methods, of the 21 index cases that initiated cluster investigation, two of the case-patients participated in the investigation and were removed from analysis. This was done in order to avoid identification bias in identification of factors associated with DENV infection.*

You said in the methodology that you tested the patients against DENV, CHIKV and ZIKV, but I only see the results for DENV, do you have the results of infected people by CHIKV and ZIKV?

*We appreciate the reviewer pointing out this discrepancy. We have added text to the Results to specify that no participants tested positive for infection with CHIKV or ZIKV.*

Would be desirable to describe a little bit the overall results of the questionnaires.

*The results of the questionnaires are reported in Tables 1 and 2.*
